# Assessing colostral and serum immunoglobulin G in alpacas using Brix refractometry and total serum protein

**DOI:** 10.1007/s11259-024-10297-0

**Published:** 2024-02-23

**Authors:** Amber K. O’Neill, Christopher E. Petzel, Joanne H. Connolly, Jane L. Vaughan, Randi Rotne

**Affiliations:** 1 School of Agricultural, Environmental and Veterinary Sciences, Charles Sturt University, Wagga Wagga, NSW 2650 Australia; 2 Gulbali Institute, Charles Sturt University, Locked Bag 588, Wagga Wagga, NSW 2678 Australia

**Keywords:** Alpaca, Camelid, Brix, Colostrum, Immunoglobulin, Passive transfer of immunity

## Abstract

The adequate transfer of passive immunity is a critical factor in neonatal development and survivability. Although well documented in the dairy and equine industries, the recognition of inadequate immunoglobulin transfer on-farm and its impact on the ability of alpaca cria to thrive is largely unknown. Colostrum samples were collected from female alpaca within 24 h of parturition by the owners and whole blood collected from cria by the investigators between 1 and 7 days of age. Direct IgG concentration of milk and serum was determined using radial immunodiffusion assay (RID) and was indirectly estimated using optical and digital Brix refractometry for total solids and clinical refractometry for total serum protein. There was a strong correlation between optical and digital Brix refractometry, and colostral IgG concentration determined by RID. There was a moderate correlation between serum IgG concentration determined by RID and total serum protein in crias. Optical and digital Brix refractometry for colostral IgG estimation and total serum protein for serum IgG estimation are reliable, accurate and easy-to-use tools that can be used on-farm by trained, competent technicians to assess a failure of passive transfer in alpacas. A pilot study at one property only was performed, due to COVID-19 travel restriction interference. Further research is required to determine the reference intervals for these tools to be practical.

## Introduction

The adequate transfer of passive immunity is critical factor of neonatal development and survivability. In species relying on colostrum intake for the passive transfer of immunity such as alpacas, the transfer of immunoglobulins, particularly immunoglobulin G (IgG) across the intestinal mucosa, through the enterocytes and into the bloodstream within the first hours of life is the physiological process that leads to that passive transfer of immunity (Garmendia et al. 1987; Bravo et al. 1997). Transfer of IgG in mammals from an immunocompetent dam to its neonate firstly provides immunity and secondly reduces the metabolic expenditure involved in generating an active immune response during the first weeks of life (Weström et al. 2020). Failure of passive transfer in alpacas ranges from 10–20% across various studies (Garmendia et al. 1987; Weaver et al. 2000a, b; Elsohaby et al. 2017) and is usually associated with risk factors such as low birth weight, evidence of prematurity/dysmaturity, dystocia, maiden dams, dams in poor body condition and/or extreme weather (Whitehead 2009).

Access to commercial camelid-specific RID kits to directly measure IgG concentrations in milk and serum is limited to a single supplier in Australia, with delivery of kits taking several weeks. Additionally, the assay runs over 24 h and results are highly dependent on the assay standard and not necessarily comparable between assays (Hutchison et al. 1995; Pinn et al. 2013).

There are a variety of indirect tools and kits available for use in clinics and on-farm, to estimate the passive transfer of IgG indirectly, including optical and digital Brix refractometry, total serum protein, serum zinc sulfate/sodium turbidity tests and serum glutamyl transferase activity (GGT) (Tyler et al. 1996; Zakian et al. 2018). The Brix refractometer is an inexpensive tool used to estimate colostral and serum IgG concentration by providing a percentage value that estimates the total solids of the sample. There are optical and digital options available which have been shown to have a high correlation to RID assays, when used to estimate serum IgG concentrations in dairy calves and foals (Bielmann et al. 2010a, b; Elsohaby et al. 2019). Total serum protein can be estimated using a clinical refractometer that measures the refractive index of a sample which is determined by its total solids (Morrill et al. 2015). Serum turbidity and GGT indirect tests are not sensitive enough to use in dairy calves or alpacas (Tyler et al. 1996; Weaver et al. 2010a, 2010b; Zakian et al. 2018).

The aim of the following study was to determine whether rapid, on-farm tools such as Brix refractometry and total serum protein were accurate compared to more costly and slower direct methods of measuring colostral and neonatal serum IgG concentration in alpacas. Optical and digital Brix refractometer assessment of colostrum and cria serum were compared to RID assays of alpaca colostrum and cria serum respectively. The study hypothesis is that the indirect methods would be in agreement with direct assays.

## Materials and methods

### Study design

A pilot study was performed where colostrum samples were obtained from dams within 14 h of parturition and serum samples were collected from crias at 1–7 days of age for IgG concentration assessment. Sample collection occurred on-farm from a property that was within 2 h of Wagga Wagga, NSW, due to restrictions of COVID-19 regulations and ease of sample collection. Initial planning with multiple properties and larger collection numbers was cancelled with the changing COVID-19 environment. Approval was obtained from the Animal Care and Ethics Committee and Human Research Ethics Committee of Charles Sturt University (20279 and H20269) prior to the study commencing.

### Alpacas included in the study design

Ten adult females, 8 male crias aged 1–7 days and 2 female crias aged 1–7 days on a Suri alpaca property in the eastern Riverina district of New South Wales were available for sample collection. An additional 3 samples were collected as part of a trial prior to the study, although were not included in the analysis. Cria were allowed to suckle from their dam with no intervention, so the quantity of colostrum that each cria received was not recorded. Seventy percent of adult females were first parity with the average parity being 1.6. Alpacas were grazed in paddocks on a private property with no supplementary feeding at the time of the study, and brought into yards at the time of sample collection. A thorough physical examination was undertaken on dams and crias allowing animals to be enrolled in the study based on the inclusion criteria (Table 1). Any animals that did not meet the inclusion criteria or reference ranges for physical parameters were excluded from the study. One female and one cria were excluded from the study.

**Table 1:** Inclusion criteria used in the study

Consent	Informed, signed consent provided by alpaca owner for inclusion of their alpaca in the study	
Age	Adult females: any age, with a cria-at-foot born within 7 days of colostrum collection	
	Cria: 1–7 days of age	
Physical parameters	Heart rate	Adult females: 50–80 bpm
		Cria: 80–140 bpm
	Respiratory rate	Adult females: 10–30 breaths/min
		Cria: 20–40 breaths/min
	Rectal temperature	Adult females: 37.5 – 39.4 °C
		Cria: 37.5 – 39.1 °C
	Packed cell volume (PCV)	Cria: 25–45% [14, 15]
	Total protein	Cria: 52–70 g/L
	Oral mucous membranes	Pink, moist, capillary refill time < 2 s
Evidence of suckling	Waxy plugs on teats not present and suckle reflex present. Cria only consuming colostrum from dam, no supplementary colostrum or milk provided. No evidence of mastitis	
Veterinary history	No administration of veterinary care the week prior to sample collection e.g. antimicrobials	
Adult female reproductive status	Cria at foot aged at 1–7 days	

All alpacas outside of the stated criteria were excluded from the study, and only those within the criteria were included for sample analysis

### Colostrum collection

Colostrum was collected at 1–14 h post-parturition consistent with the previous work in bovids and camelids (Garmendia et al. 1987; Miller 2013; Weaver et al. 2000a, b). Each cria remained with its dam during colostrum and blood collection to reduce stress caused by separation, although each pairing was separated from other animals for safety of both alpacas and handlers. A physical examination was undertaken, the udder palpated to identify any abnormalities (heat, swelling, masses) and the presence or absence of a waxy plug in the end of each teat was recorded. The collector wore disposable nitrile gloves that were changed between each alpaca. The udder was wiped before and after milking with a new 70% alcohol-based teat wipe to prevent contamination of the colostrum sample from the udder skin. Colostrum was manually milked from each of the four teats, the total amount equaling approximately 10 mL per female, where possible. Colostrum was collected into a sterile 50 mL container that was labelled with an identification number and date and refrigerated in a portable fridge at 4 °C for transport from farm to laboratory. Samples were then immediately frozen at -20 °C until IgG analysis was undertaken. Refractometry was not performed on-farm at the time of collection as RID analysis could not be performed concurrently due to laboratory requirements. Additionally using a fresh colostrum sample for refractometry and a frozen sample for RID analysis may have added uncertainty to the results and was avoided.

### Colostrum laboratory analysis

Colostrum was thawed to 20 °C in a warm water bath immediately prior to analysis (Morrill et al. 2015). A digital refractometer HI 96811 (Hanna Instruments, Europe, Romania) and E-Line automatic temperature compensation (ATC) range optical Brix refractometer (Bellingham + Stanley, Tunbridge Wells, Kent) was used to measure the total solids in the colostrum. The digital Brix refractometer was calibrated before each sample analysis by filling the sample well with deionised water to ensure the reading was accurate. The sample well was then dried with a Kimwipe® (Kimberley-Clark, Australia) and a few drops of colostrum were placed using a pipette in the sample well until filled completely and the % Brix recorded. The optical Brix refractometer was calibrated before each use by opening the illuminator flap, pipetting a few drops of deionised water onto the prism, closing the illuminator flap and adjusting the calibration screw to ensure the Brix % read as ‘0’. The prism was wiped using a Kimwipe® and a pipette used to place a few drops of colostrum onto the prism. The illuminator flap was closed and the Brix % recorded. Camelid RID analysis kits (Radial Immunodiffusion Test for Quantification of Camelid IgG in Serum or Plasma®, Triple J Farms, Bellingham, Washington, USA) were used for direct IgG assessment. Each kit included a single 24-well agrose gel plate containing camelid IgG antiserum, and three reference sera samples of known concentration (203 mg/dL, 1452 mg/dL and 2851 mg/dL). The test kits were stored at 4 °C prior to use and removed from the refrigerator 30 min prior to filling the wells, as directed by the manufacturer’s instructions. Five uL of thawed colostrum or reference sera was delivered into each well using a digital caliper (Calibra® digital 822 1–10 uL and Diamond 10 uL pipette tips). Each test was duplicated. The plates were incubated at 22 °C for 24 h to achieve end point readings. The precipitin ring zone diameters were measured for each sample following incubation using a digital caliper (Craftright® digital vernier caliper 150 mm, Bunnings Group Australia). Colostrum samples that exceeded the top reference sample diameter were diluted and the assay was repeated. Of the 10 colostrum samples analysed, 4 were diluted 1:10 in physiological saline and 6 were diluted 1:15 in physiological saline to allow accurate measurement. The zone diameters of the reference samples were graphed with IgG concentration (mg/dL) plotted on the x-axis and zone diameters squared (mm) on the y-axis. A line of best fit was generated, and the standard graph was used to determine the IgG concentration (mg/dL) of the unknown colostrum samples, based on each zone diameter squared. Finally, the IgG concentration of each original undiluted colostrum sample was calculated with application of the appropriate dilution factor (for example, colostrum samples diluted 1:10 were multiplied by 10 after analysis to reveal the IgG concentration of the original, undiluted colostrum sample).

### Blood collection and analysis

Blood collection from cria occurred at 1 to 7 days of age, as it has been shown that serum IgG concentrations increase until 24 h of age and then plateau for 7 days before steadily decreasing (Garmendia et al. 1987; Bravo et al. 1997; Miller 2013; Weaver et al. 2000a, b). All cria were observed to suckle from their dam within 3 h of birth. Each cria was restrained by wrapping it in a towel in the cush position (natural sitting position) and placing on a table with the neck nearest the edge of the table. The handler supported the head of the cria in a relaxed position to allow the right jugular vein to be palpated at the mid-lower third of the neck by the collector. A sterile 22-gauge, 1.9 cm needle and 5 mL syringe was used for each blood collection. Fleece was clipped using electric clippers from the venipuncture site over the right jugular vein then cleaned with chlorhexidine and swabbed with a 70% alcohol cotton ball. Five mL of blood was collected from each cria from the right jugular vein, of which 4 mL was placed into a 4 mL plain blood tube (BD Vacutainer®, Plymouth, UK) and the remaining 1 mL of blood placed into a 1 mL heparin blood tube (Sarstedt Inc, Numbrecht, Germany). Heparin blood tubes were inverted gently ten times to ensure adequate mixing with anticoagulant. Manual pressure was applied to the vein for 60 s after the needle was removed to preserve the vein and minimise haematoma formation. Each cria was then returned to its dam and observed to ensure it suckled.

Whole blood was left to clot at room temperature for 20 min and then refrigerated for a maximum of 5 h before centrifugation at 2500 rpm for 10 min. Serum was collected from the blood tube using a pipette and placed into a new plain blood tube and stored at -20 °C until analysed for IgG concentration. The remaining blood clot was frozen and stored separately, but not used for analysis.

Heparinised blood was used to measure packed cell volume (PCV) to assess hydration status, with PCV > 45% classified as dehydrated. The whole blood samples were centrifuged in a microhematocrit capillary tube at 10,000 rpm for five minutes and the PCV measured using a PCV reader and then recorded.

### Laboratory analysis

At the time of sample analysis serum was thawed in a 20 °C water bath. The digital Brix refractometer was calibrated before each sample analysis in a similar manner to colostrum analysis, and then the serum Brix % recorded. Total serum protein was measured using a clinical refractometer. Deionised water was placed onto the prism before each sample analysis to calibrate and ensure the reading was accurate. A few drops of serum were placed on the prism using a pipette and the cover plate secured. Total serum protein was estimated by observing the reading through the eye piece.

Camelid RID analysis kits were used for direct IgG assessment, as for colostral IgG concentration. Four of the serum samples required dilution and were diluted 1:2 in physiological saline. All samples were duplicated for analysis. The zone diameters of the reference samples were graphed, a line of best fit generated and the unknown serum sample IgG concentrations determined, the same as for colostrum sample analysis. The final IgG concentration of each serum sample was determined with application of an appropriate dilution factor.

### Data analysis

Packed cell volume was measured within 5 h of collection to assess hydration status (in addition to mucous membrane characteristics; moisture, colour, capillary refill time) and results were recorded.

Statistical analysis was performed using R: A Language and Environment for Statistical Computing (version 4.0.3). Probability (*P*) values < 0.05 were considered statistically significant. Pearson’s correlation coefficients of zero were considered as having no linear relationship (Kirch 2008). Correlation coefficients ± 30 from zero were considered as having a weak linear relationship. Those > 50 but < 70 were identified as having a moderate correlation and correlation coefficients > 70 were considered as having a strong linear relationship. For assessment of sensitivity, cut-off values for Brix refractometry and colostral and serum IgG concentrations were used. For colostrum, high quality was defined as having an IgG concentration > 25,000 mg/dL and low quality having < 25,000 mg/dL and a Brix cut-off value of 32% was selected.

Previous dairy research has identified that the r-values for IgG assessment using refractometry on colostrum and neonatal serum is 0.75 and 0.85 respectively (Bielmann et al. 2010a, b; Thornhill et al. 2015). A power analysis of correlation sample size (Hulley et al. 2013) was undertaken using the bovine r-values, and standard values for Type I error (α = 0.05) and Type II error (β = 0.2). The standard power analysis identified that a minimum of 10 animals is required for the colostrum IgG assessment and a minimum of 8 animals is required for the serum IgG assessment. An intermediate power analysis of correlation sample size was also considered with α = 0.05 and β = 0.1 resulting in 14 & 10 animals being needed for the colostrum and serum IgG assessment respectively. It was decided the sample size of 10 animals was acceptable as it met the minimum sample size for the standard and intermediate power analyses of correlation sample size for colostrum and serum IgG assessment respectively.

For this study, inadequate passive transfer of immunity was defined as crias having a serum IgG concentration < 10 g/L based on recent studies (Pinn et al. 2013; Weaver et al. 2000a, b).

The optical Brix refractometer upper limit was 32%, so those samples that were read as > 32% were assigned a measurement of 32% for statistical analysis.

## Results

### Colostral IgG concentration

The RID IgG concentrations are shown in Table 2. The study population characteristics of the dam and cria and estimation of colostral IgG concentration using optical and digital Brix refractometry are also shown in Table 2.

**Table 2:** Study population characteristics and results of colostral IgG analysis

Female identity	Female age (years)	Cria age (hours)	RID [IgG] (g/L)	Optical Brix %	Digital Brix %
4	9	6	85.8	16	14.2
5	6	14	66	18	18
7	3	2	329.6	> 32	41.2
8	11	1	257.6	> 32	41.6
9	8	4	257.6	> 32	35.5
10	8	1	404.8	> 32	37.1
11	11	3	358.4	> 32	41.1
12	7	1	340.8	22	22.5
13	5	2	205.7	32	32.3
14	6	3	235.2	31	31.6

### Comparison of Brix refractometry and RID for colostral IgG concentration

**Fig. 1:**
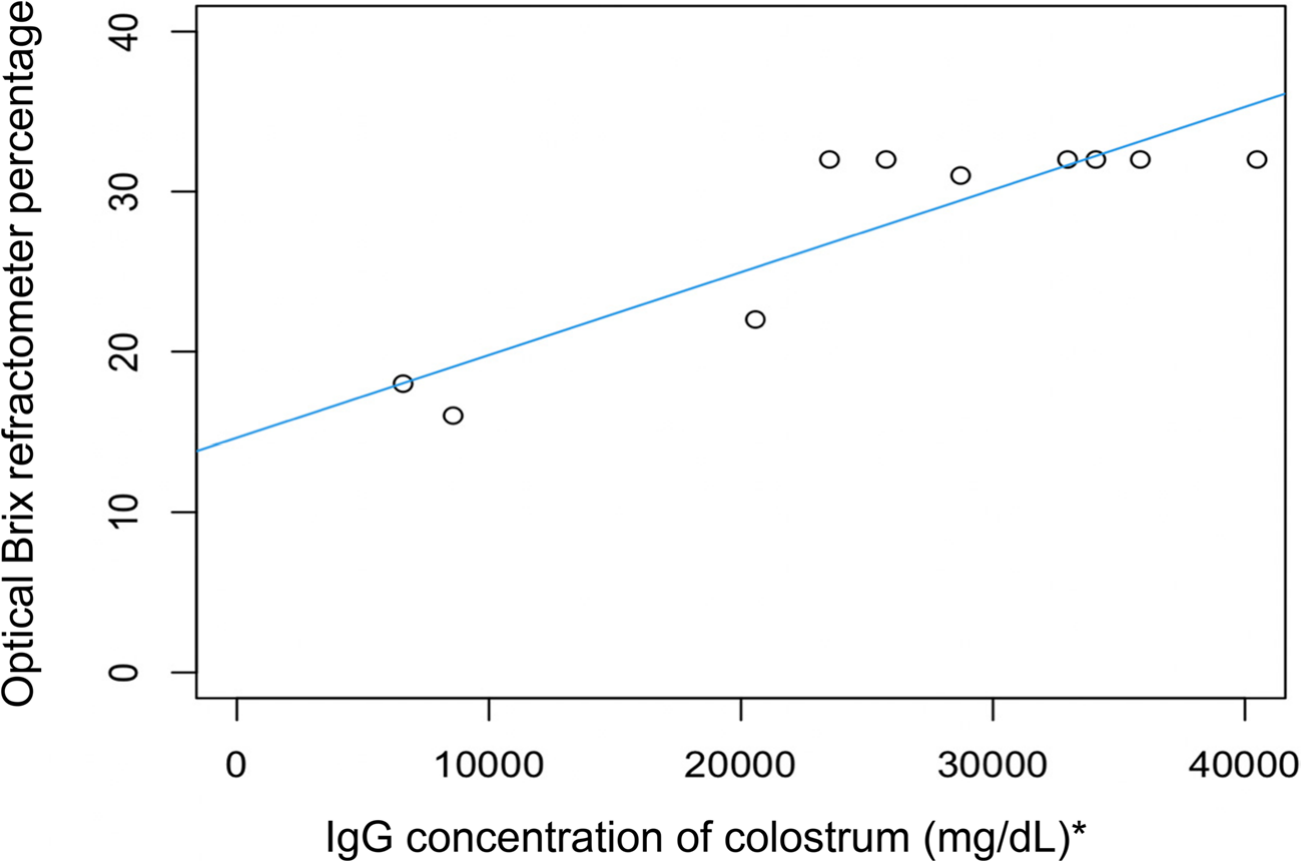
Linear correlation between colostrum IgG concentration (measured using RID) and optical Brix refractometer percentage for colostrum (adjusted R2 = 0.77, *P* = 0.0005, *n* = 10). (* To convert mg/dL to g/L divide by 100)

The relationship between colostral IgG concentration (determined by RID) and optical Brix refractometry is shown in Fig. 1. The blue line is the line of best fit; a straight line that best represents the relationship between the data points plotted. There was a strong linear correlation (correlation coefficient 0.89) and the correlation was statistically significant (simple linear regression model, *P* = 0.0005 and adjusted R-squared = 0.77, *n* = 10) (Kirch 2008).

**Fig. 2:**
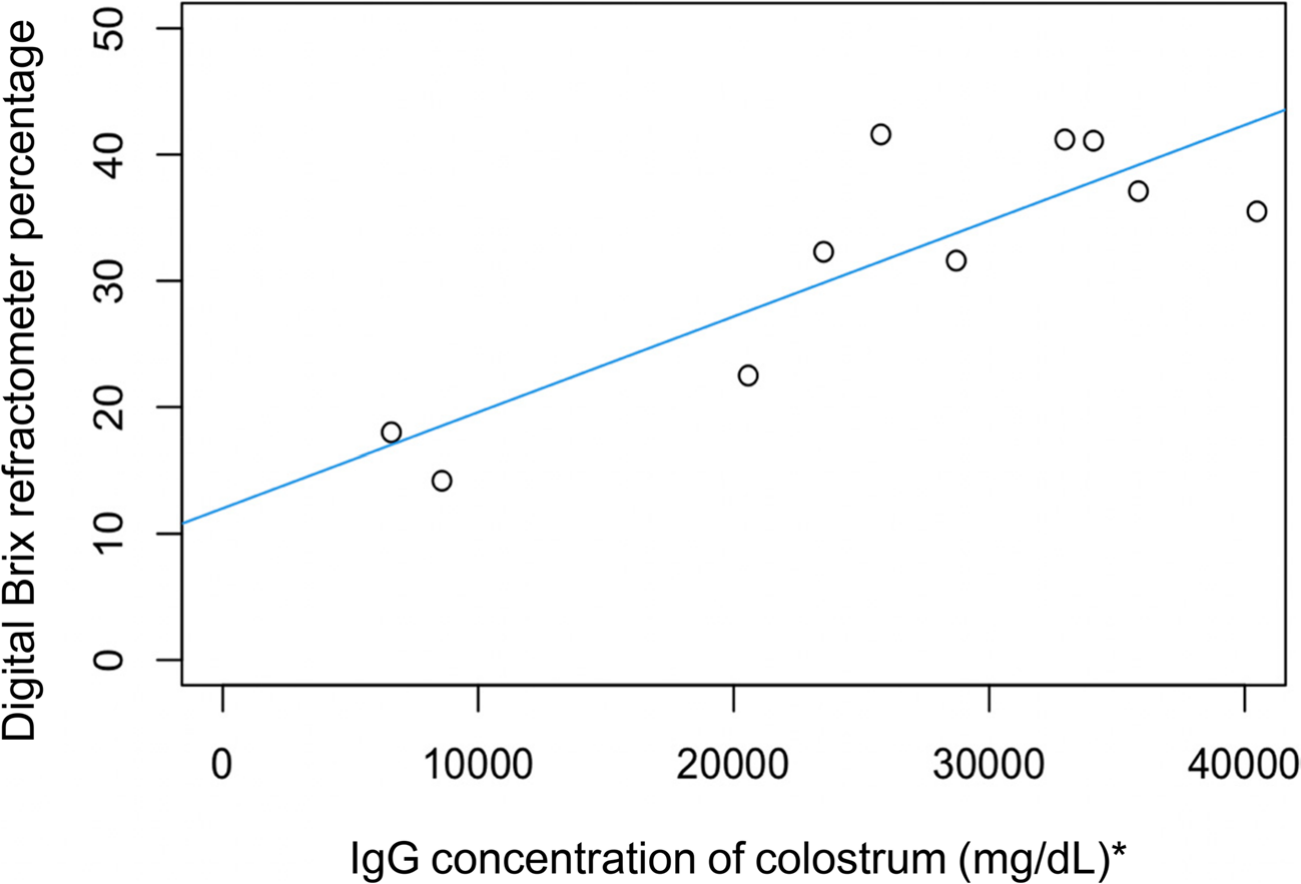
Linear correlation between colostrum IgG concentration (measured using RID) and digital Brix refractometer percentage for colostrum (adjusted R2 = 0.70, *P* = 0.002, *n* = 10). (* To convert mg/dL to g/L divide by 100)

The relationship between colostral IgG concentration (determined by RID) and digital Brix refractometry is shown in Fig. 2. A strong linear correlation was present (correlation coefficient 0.86) and the correlation was statistically significant (simple linear regression model, *P* = 0.002 and adjusted R-squared = 0.70, *n* = 10).

**Fig. 3:**
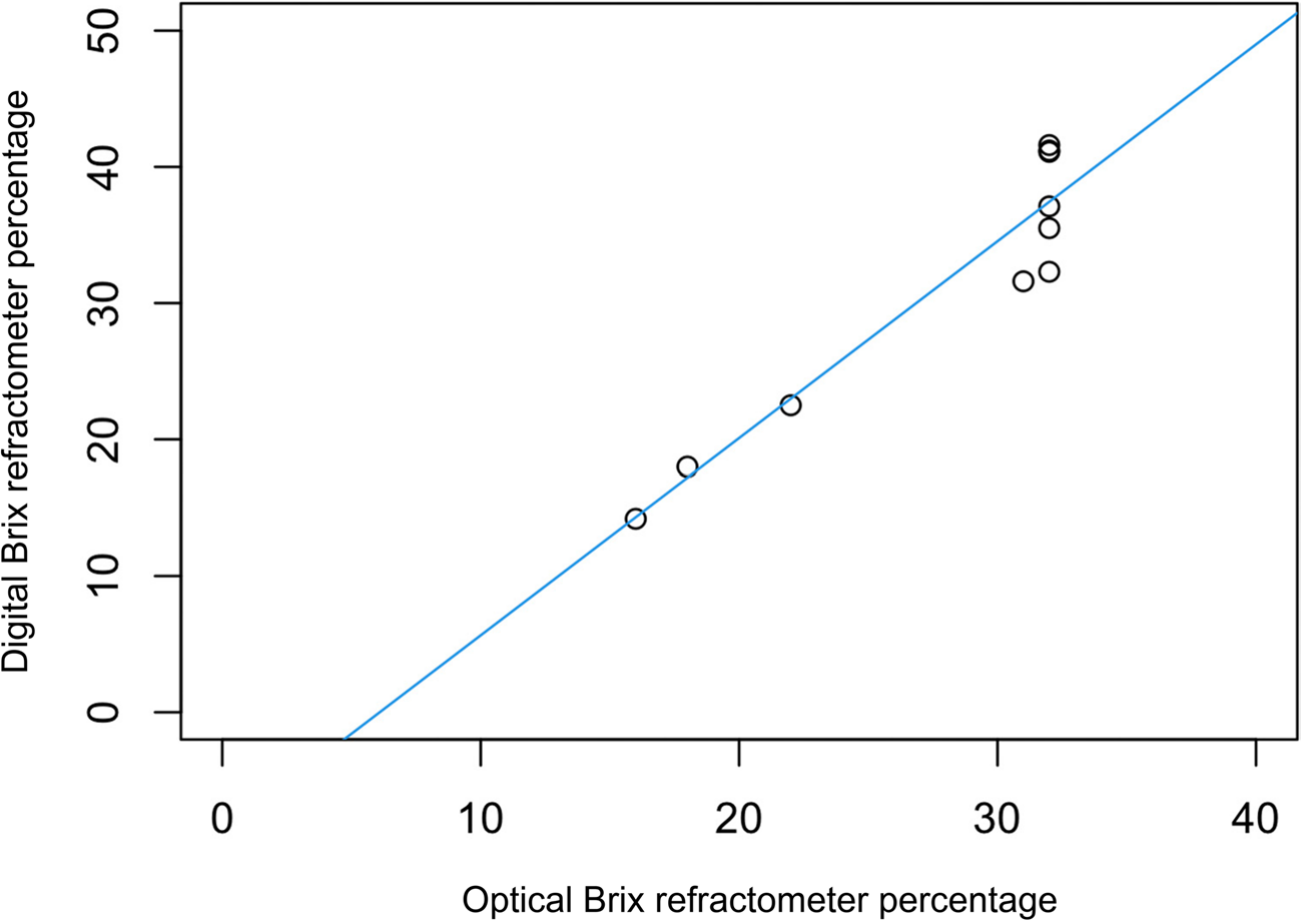
Relationship between optical and digital Brix refractometer percentage for colostrum

The relationship between colostral IgG concentration estimated by optical Brix refractometry and colostral IgG concentration estimated by digital Brix refractometry is shown in Fig. 3. There was a strong linear correlation (correlation coefficient 0.95) and the correlation was statistically significant (simple linear regression model, *P* = 0.00003 and adjusted R-squared = 0.88, *n* = 10).

### Serum IgG concentration

The IgG concentration determined by RID or estimated by optical and digital Brix refractometry and total serum protein of each cria serum is presented in Table 3.

**Table 3:** Results of serum IgG analysis, including age of cria, IgG determined by RID, Brix percentages, PCV for hydration assessment and total serum protein

Cria identity	Age (days)	RID [IgG] (g/L)	% Brix		PCV (%)	Total serum protein (g/L)
			Optical	Digital		
4	5	23.0	7.8	7.79	38	6.2
5	5	23.1	7.4	7.4	38	6.0
7	5	30.0	8.2	8.3	40	6.6
8	5	26.4	7.4	7.1	38	6.0
9	1	34.2	7.8	8.0	33	6.4
10	1	47.7	8	8.4	34	6.6
11	6	11.4	7.6	7.7	45	6.2
12	2	21.3	7.0	6.6	34	5.4
13	1	17.7	6.8	5.0	34	5.4
14	6	17.9	7.4	7.3	32	6.0

**Fig. 4:**
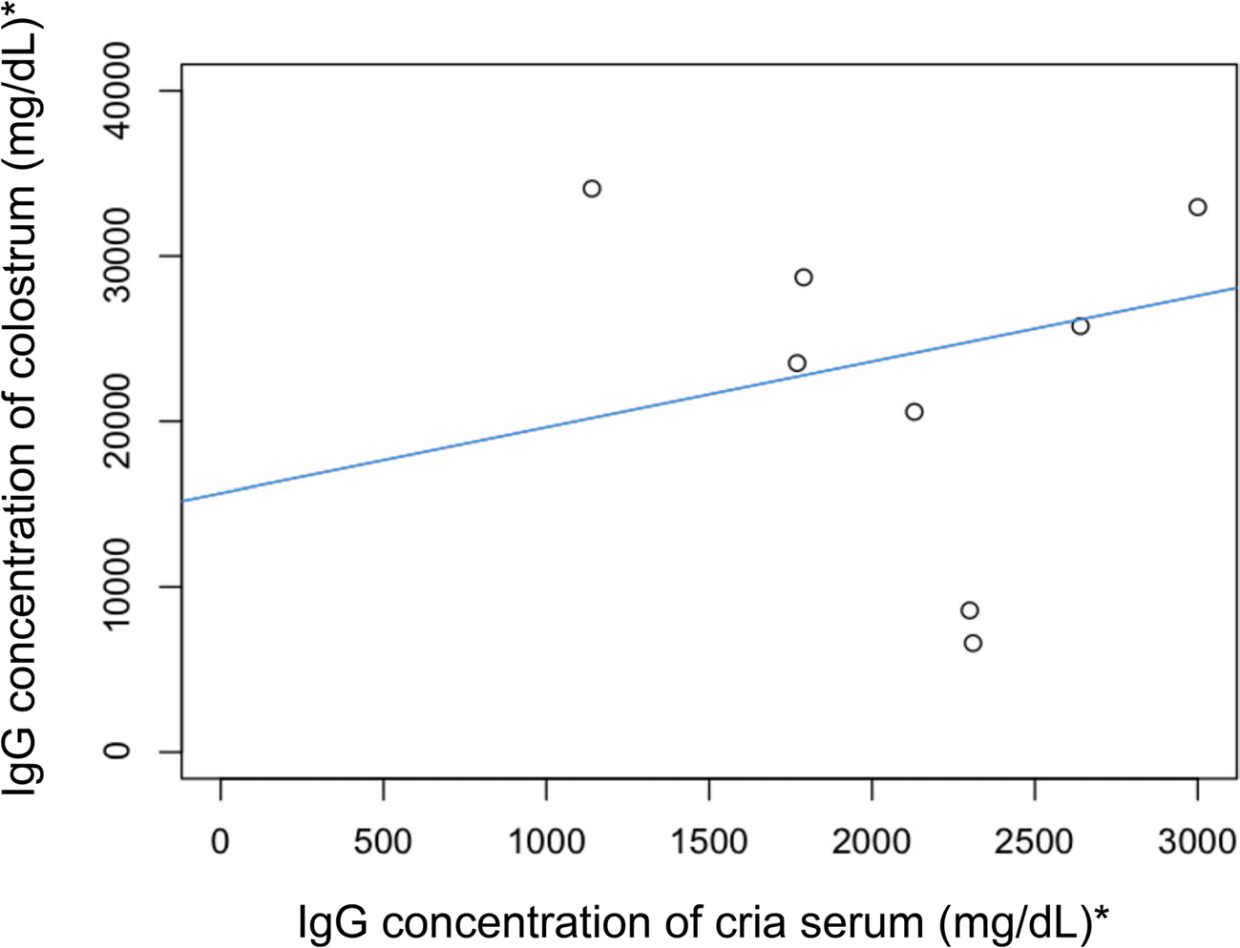
Linear correlation between cria serum and dam colostrum IgG concentration, determined by RID (adjusted R2 = 0.02, *P* = 0.31, *n* = 8). (* To convert mg/dL to g/L divide by 100)

Comparison of Brix refractometry, total serum protein and RID for serum IgG concentration A moderate linear correlation was present between RID-determined serum and colostral IgG concentration (Fig. 4) (correlation coefficient 0.36, simple linear regression model, *P* = 0.31, adjusted R-squared = 0.02, *n* = 8).

**Fig. 5:**
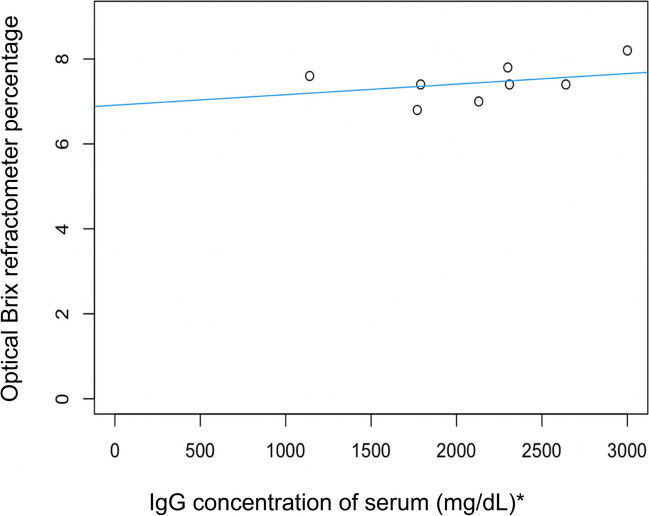
Linear correlation between cria serum IgG concentration (measured using RID) and optical Brix refractometer percentages for serum (adjusted R2 = 0.26, *P* = 0.08, *n* = 8). (* To convert mg/dL to g/L divide by 100)

The relationship between serum IgG concentration (determined by RID) and optical Brix refractometry is shown in Fig. 5. There was a moderate linear correlation (correlation coefficient 0.59, simple linear regression model, *P* = 0.08, adjusted R-squared = 0.26, *n* = 8).

**Fig. 6:**
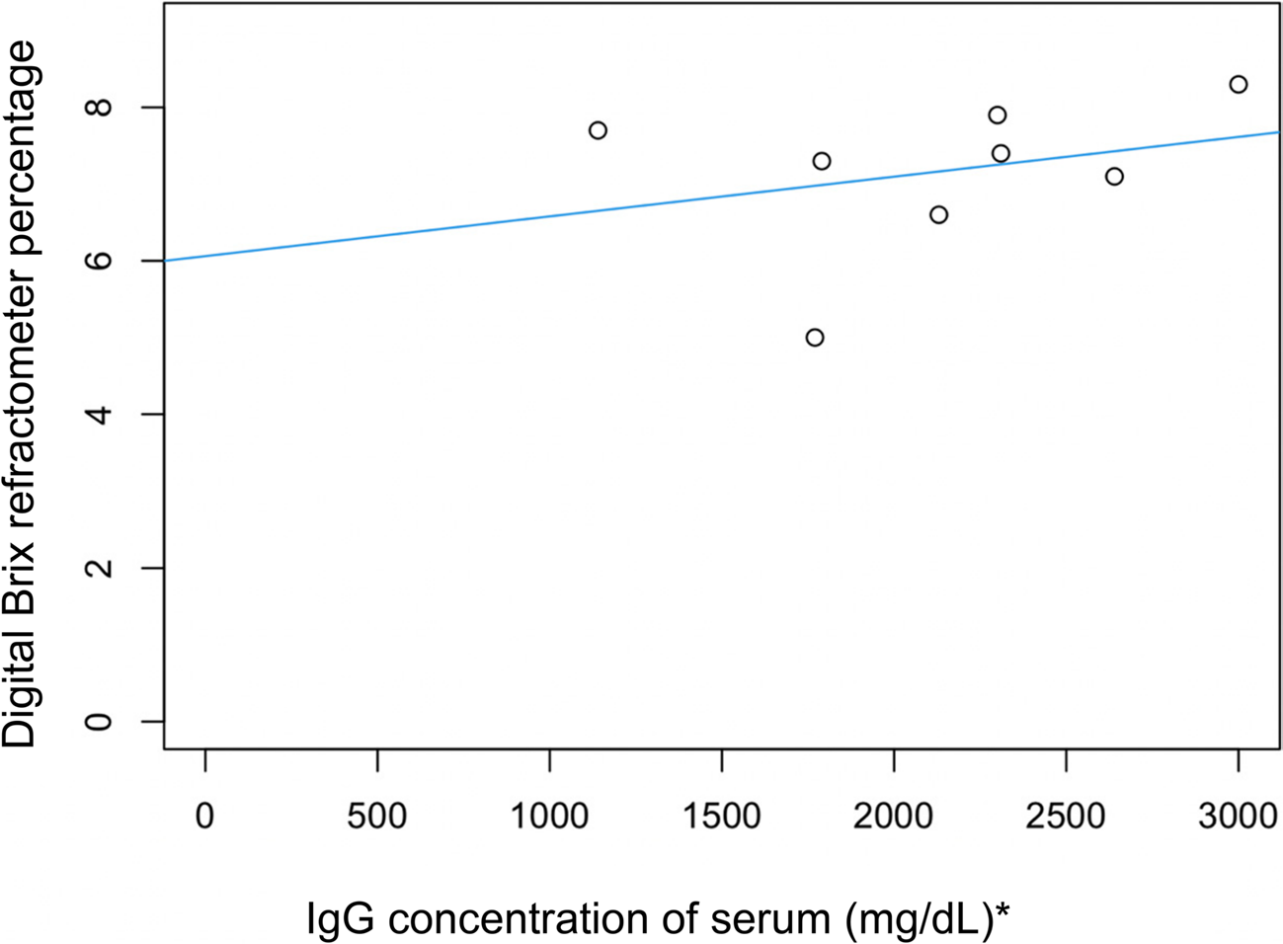
Linear correlation between cria serum IgG concentration (measured using RID) and digital Brix refractometer percentages for serum (adjusted R2 = 0.19, *P* = 0.12, *n* = 8). (* To convert mg/dL to g/L divide by 100)

The relationship between serum IgG concentration (determined by RID) and digital Brix refractometry is shown in Fig. 6. There was a moderate linear correlation (correlation coefficient 0.53, simple linear regression model, *P* = 0.12, adjusted R-squared = 0.19, *n* = 8).

**Fig. 7:**
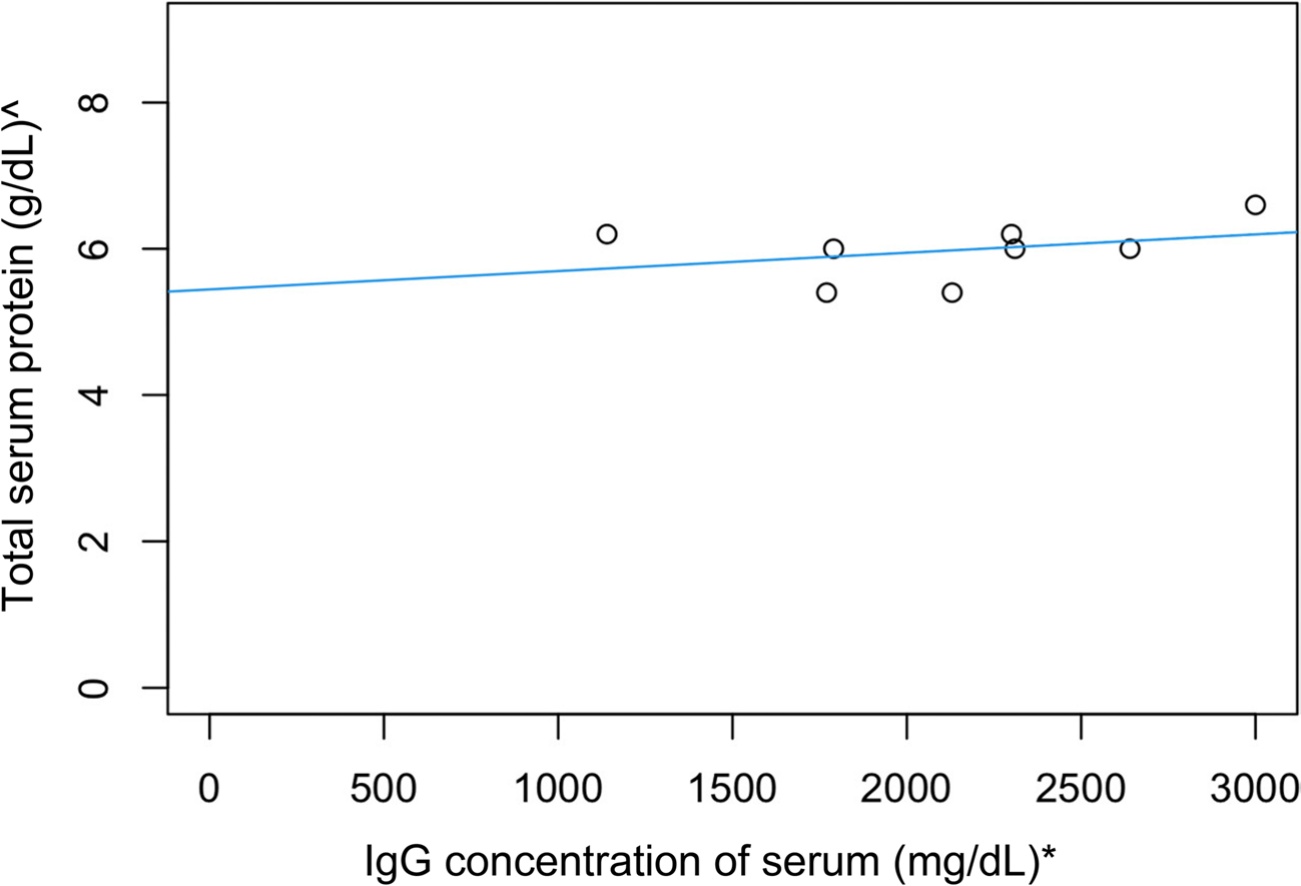
Linear correlation between cria serum IgG concentration (measured using RID) and total serum protein measured using a clinical refractometer (adjusted R2 = 0.29, *P* = 0.06, *n* = 8). (* To convert mg/dL to g/L divide by 100)

The relationship between serum IgG concentration (determined by RID) and total serum protein is shown in Fig. 7. There was a moderate linear correlation (correlation coefficient 0.61, simple linear regression model, *P* = 0.06, adjusted R-squared = 0.29, *n* = 8).

**Fig. 8:**
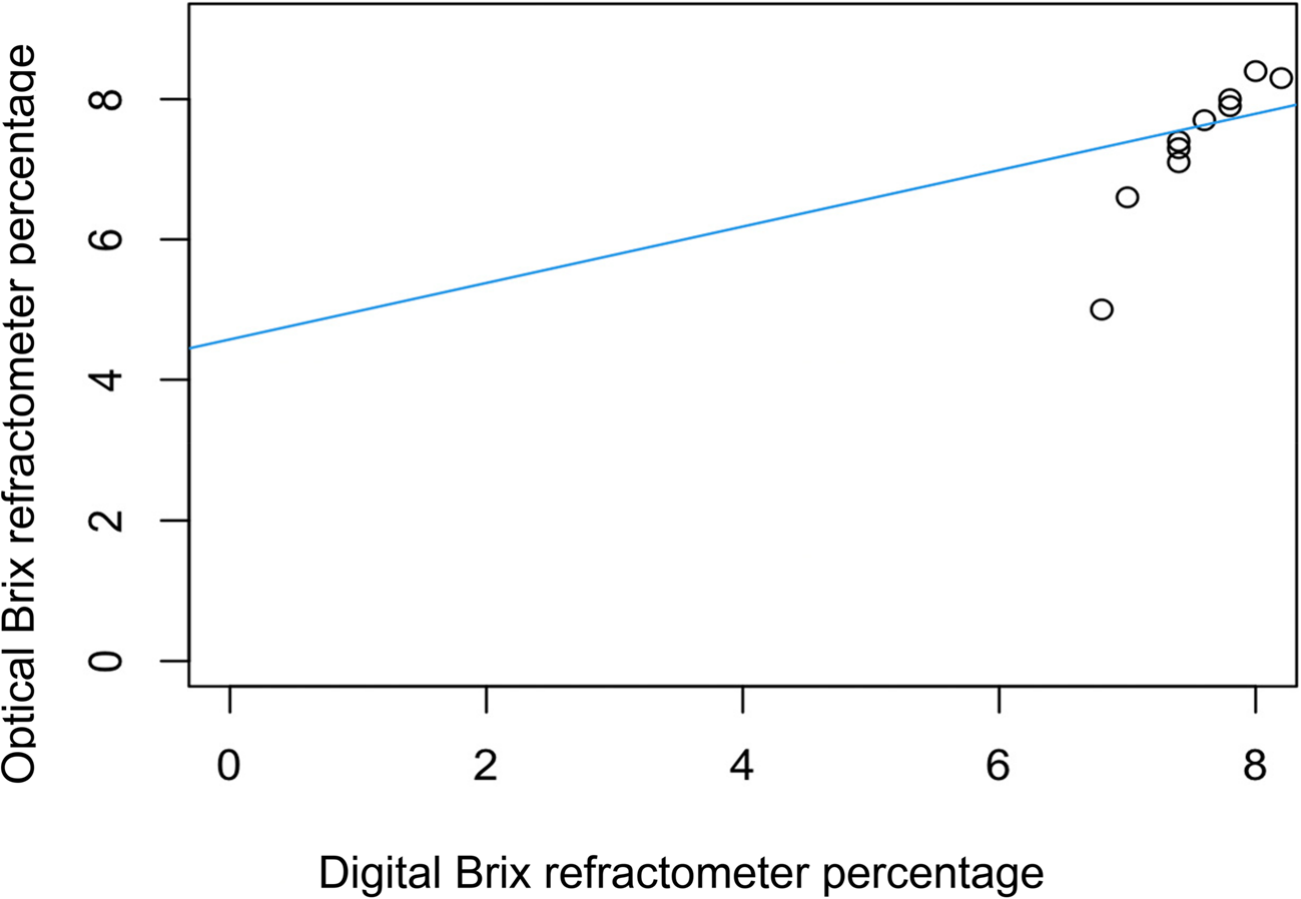
Linear correlation between optical and digital Brix refractometer percentages for cria serum (adjusted R2 = 0.85, *P* = 0.00009, *n* = 10). (* To convert g/dL to g/L multiply by 10)

The relationship between optical and digital Brix refractometry for estimation of serum IgG concentration is shown in Fig. 8. A strong linear correlation was present (correlation coefficient 0.93, simple linear regression model, *P* = 0.00009, adjusted R-squared = 0.85, *n* = 10).

## Discussion

This study supported the hypothesis that on-farm tools such as Brix refractometry and total serum protein can be used to estimate colostral and serum IgG in alpacas, respectively. Optical and digital Brix refractometry was found to be an appropriate and accurate tool for assessing colostral IgG concentration (Figs. 1 and 2), and optical/digital Brix and total serum protein was moderately correlated with serum IgG concentration in alpacas (Figs. 5, 6 and 7).

There were significant linear correlations between digital and optical brix refractometer percentages of dam colostrum (r = 0.95; Fig. 3) and cria serum (r = 0.93; Fig. 8), indicating no advantage of one refractometer over the other. Similarly, Elsohaby et al. (2017) found a high correlation (r = 0.99) between digital Brix and optical serum total protein refractometry.

The assessment of optical and digital Brix refractometry, compared to RID analysis for colostrum both resulted in a strong correlation with statistical significance, shown by a correlation coefficient > 85 and a *P* value < 0.05. To the authors’ knowledge Mößler et al. (2022) is the only other research that has used Brix refractometry in assessing colostral IgG in alpacas. Bravo et al. (1997) found the mean IgG concentration of alpaca colostrum at parturition was 217.9 ± 7.9 g/L (range 176.5–284.4; *n* = 15). In our study, 220 g/L IgG equated to optical and digital Brix around 32% (Table 2). It was not possible to determine a Brix % cut-off point for colostrum IgG concentration likely to result in failure of passive transfer as all crias had IgG concentrations > 10 g/L so received adequate quality and quantity of colostrum on their first day of life. The cut-off percentage where neonates are likely to suffer failure of passive transfer in dairy cattle and horses vary from Brix 18% to 23% respectively (Morrill et al. 2015; Mößler et al. 2022), although IgG concentration of camelid colostrum is reported to be substantially higher than other species (Mößler et al. 2022).

There was no significant linear correlation between dam colostrum IgG concentration and cria serum IgG concentration (Fig. 4). There are many variables that determine neonatal ability to suck colostrum in a timely fashion and achieve adequate passive transfer. Regardless of cause, delay in suckling colostrum will result in reduced absorption of immunoglobulins and increase risk of failure of passive transfer (Fowler 2010).

The assessment of optical and digital Brix refractometry and total serum protein by optical refractometry, compared to RID analysis for serum resulted in a moderate correlation of all indirect tools with the limitation of small sample size (Figs. 5, 6 and 7). Previous research indicates that failure of passive transfer in camelids has occurred if the cria serum IgG concentration at 1–2 days of age is < 10 g/L (Bravo et al. 1997; Wernery 2001; Weaver et al. 2000a, b). In a group of 169 mixed-age alpacas, the cut-off equivalents for serum IgG concentration < 10 g/L were digital Brix ≤ 8.8% and serum total protein ≤ 50 g/L using an optical handheld refractometer (Elsohaby et al. 2017). In our study, all crias had serum IgG concentrations > 10 g/L, and all total serum proteins were > 50 g/L but optical and digital Brix measurements were < 8.8% (Table 3). The moderate correlation between digital/optical Brix and serum protein refractometry with serum IgG concentrations is promising. While the findings were not statistically significant the small sample numbers could have contributed to these results. The significant and strong correlations between refractometers for colostrum and serum tests suggest that further study utilising larger numbers would be beneficial. However, the use of a refractometer for the assessment of serum IgG concentration has limitations on-farm, as blood must be collected by a competent, trained person such as a veterinarian and then centrifuged.

## Conclusions

The use of optical and digital Brix refractometry to assess colostral IgG concentration, and total serum protein to assess serum IgG concentration, allows alpaca owners and their veterinarian to estimate adequacy of passive transfer of immunity soon after birth when factors such as low birth weight, evidence of prematurity/dysmaturity, dystocia, dams of low parity and /or poor body condition, and/or exposure to extreme weather will increase the risk of failure of passive transfer. This will enable intervention measures to be implemented early if required to improve the survivability of crias. Although the definition of poor quality colostrum is unknown in alpacas, the results of this paper confirm that on-farm tools can be utilised to optimise passive transfer of immunity. Further research into the normal parameters of serum and colostral IgG concentration in large numbers of crias and their dams, including both Suri and Huacaya breeds, across many farms will better enable these tools to play a significant role in neonatal cria care.

## Data Availability

Data available on request. The data presented in this study are available on request from the corresponding author.
